# Minimizing Skin Scarring through Biomaterial Design

**DOI:** 10.3390/jfb8010003

**Published:** 2017-01-21

**Authors:** Alessandra L. Moore, Clement D. Marshall, Michael T. Longaker

**Affiliations:** 1 Division of General and Gastrointestinal Surgery, Brigham and Women’s Hospital, Boston, MA 02115, USA; amoore4@stanford.edu; 2 Department of Surgery, Stanford University School of Medicine, Stanford, CA 94305, USA; cmarshal@stanford.edu; 3 Institute of Stem Cell Biology and Regenerative Medicine, Stanford University, Stanford, CA 94305, USA

**Keywords:** wound healing, biomaterials, scar, tissue engineering

## Abstract

Wound healing continues to be a major burden to patients, though research in the field has expanded significantly. Due to an aging population and increasing comorbid conditions, the cost of chronic wounds is expected to increase for patients and the U.S. healthcare system alike. With this knowledge, the number of engineered products to facilitate wound healing has also increased dramatically, with some already in clinical use. In this review, the major biomaterials used to facilitate skin wound healing will be examined, with particular attention allocated to the science behind their development. Experimental therapies will also be evaluated.

## 1. Introduction

Despite the past decade’s rapid increase in knowledge concerning cutaneous wound healing, issues of dysfunctional wound healing in the U.S. continue to be a major fiscal and physical burden to patients. The number of annual surgical operations now exceeds 70 million, increasing the number of iatrogenic acute wounds in the U.S. The average number of patient comorbidities is increasing as well [1,2]. With the population both aging and becoming more obese, problems with wound healing related to surgery have cost the U.S. economy an estimated $25 billion annually [1,2]. In the case of chronic venous stasis ulcers alone, 2 million work days per year are lost due to treatment requirements. Overall, venous stasis ulcers cost American patients $2 billion in lost wages. Additionally, the psychological effects of chronic pain due to wounds has a pronounced negative impact on patient mood, relationships, and mobility [1]. In 2013, the global wound care product market generated $7.53 billion in revenue, a mark that is expected to generate up to $10.16 billion U.S. by 2020 [1,2]. With the need for wound-healing products ever increasing, it is important to review their efficacy and the science involved behind product development.

Wound-healing products typically aim to either reduce scarring, decrease the time of wound healing, or reduce inflammation. With knowledge of the adult wound-healing mechanism, expedited wound healing is often accomplished through intervening in one or several of the phases of wound healing. Initial efforts to influence wound healing targeted mostly cell signaling cascades, growth factors, or cell types. However, this proved to be minimally fruitful in human patients, in whom the process of wound healing is often redundant, and multiple cell types may possess similar abilities [3,4,5,6,7]. For instance, the well-documented failure of Avotermin (recombinant transforming growth factor-β3 (TGF-β3)) to produce its phase II endpoints in clinical trials highlighted the need for wound healing products that intervene at multiple levels in the wound healing mechanism, or, the need to develop novel approaches that influence wound healing altogether [8].

As such, the new generation of wound-healing products attempt to recapitulate the three-dimensional microenvironment of normal tissue [9,10]. Through the use of natural and synthetic biomaterials, improved wound healing can be accomplished with the provision of an extracellular matrix that is superior to that provided by platelets and fibroblasts alone. With further study, the importance of the extracellular matrix (ECM) in providing cellular cues to differentiate, polarize, and migrate has expanded, emphasizing its potential as a vulnerary target [11,12,13,14,15,16]. Additionally, the study of the remarkable phenomenon of fetal scarless wound healing has identified new cellular targets which can be incorporated into biomaterial design [11,17].

In this article, we will review our current knowledge of wound healing and then describe the major biomaterials currently in use as wound-healing adjuncts. We will then discuss future therapeutic targets. Our hope is that this review can serve as a critical reference to aid clinicians and scientists in selecting, modifying, and combining treatment modalities for the purposes of research and clinical use.

## 2. Wound-Healing Overview

The process of wound healing involves fundamental and coordinated interactions between damaged tissue, recruited inflammatory cells, and local fibroblasts. To facilitate understanding, it is described in three distinct phases (though it is more appropriately described on a continuum). Each phase is distinguishable by the type and number of recruited inflammatory cells. The initial phase of wound healing begins with the establishment of tissue injury. The initial injury damages the vascular endothelium and exposes the basal lamina. As a result, blood constituents extravasate into the injured space, activating the coagulation cascade via tissue factor and the extrinsic pathway [18,19]. Along with the activation of hemostasis, inflammatory cytokines are released, resulting in further capillary leakage as well as recruitment of inflammatory cells (Table 1). The influx of predominantly neutrophils initiates the process of sterilization, re-epithelization, and eventual wound remodeling. Additionally, the formation of platelet plugs serve as an ECM substitute, providing a scaffold for inflammatory cells to enter the wound bed [2]. The platelet plug also serves as a mechanical barrier to the external environment, comprised of proteins like fibronectin, vitronectin, thrombospondin, and SPARC (secreted protein, acidic and rich in cysteine), which will similarly aid in the recruitment of inflammatory cells and bind other ECM molecules together [20]. The ECM will ultimately serve as an important shield, growth factor reservoir, and matrix to modulate wound healing with substances like hyaluronic acid, proteoglycans, glycosaminoglycans, and collagen [20]. Interestingly, it is the ECM which is responsible for the initial recruitment of inflammatory cells through proteolytic cleavage of ECM-bound growth factors and cell-signaling molecules, bringing circulating inflammatory cells into the wound [21,22,23,24,25].

The first cells to arrive at the site of injury are polymorphonuclear cells such as neutrophils. These cells scavenge damaged tissue and degranulate to recruit circulating monocytes. In the first phase of wound healing, monocytes entering the wound from circulation quickly differentiate into macrophages once in tissue. Through the release of cytokines (such as interleukin (IL)-1, -8, and -10; fibroblast growth factor (FGF); and endothelial growth factor (EGF)), macrophages initiate the formation of granulation tissue. Granulation tissue then develops through a combination of angiogenesis and migration of fibroblasts and endothelial precursor cells onto the wound surface [2,12,18].

Interestingly, neither platelets nor neutrophils are obligatory for normal wound healing in experimental animals. This is in sharp contrast to humans, in whom neutropenia represents a profound healing defect [1,2,12,18,19]. Wound healing in mice can also occur in the absence of a platelet plug, and genetic impairment or functional reduction of neutrophils causes no appreciable delay in wound healing [28]. This could be the result of redundancies in the inflammatory pathway, since many of the cell types attracted to the site of injury release similar cytokines. Ultimately, macrophages and fibroblasts may play the pivotal role in wound healing, with macrophages being responsible for the transition from inflammatory to proliferative healing [2,28].

A notable discovery in the study of wound healing includes that macrophages are obligatory for normal wound healing [37]. Macrophages, which are the differentiated tissue resident version of circulating monocytes, function as antigen-presenting cells, scavengers of cellular debris, and also helper cells that regulate the response of fibroblasts by providing cues like IL-10 and vascular EGF (VEGF) to initiate collagen deposition and remodeling [38]. Also, macrophages are the only recruited circulating cell present in multiple phases of wound healing, but with distinctly different functions. In the inflammatory phase of wound healing, M1 macrophages, also recognized as the “proinflammatory” macrophages, recruit additional inflammatory cells through the release of cytokines like IL-6 and tumor necrosis factor-α (TNF-α), and scavenge damaged tissue and debris [39]. M2 macrophages, which appear in later phases of wound healing such as the proliferative phase, are considered “pro-regenerative”. Now, a variety of additional macrophage subtypes are understood, with subtle differences in functional ability [40].

An important signaling cascade in the macrophage response to wounds is that of CXCL12 (C-X-C motif chemokine ligand 12, also known as stromal cell-derived factor 1 (SDF1)). This ECM-bound protein, once cleaved by mixed metalloproteinases (MMPs) in wounds, functions to aid in the recruitment of inflammatory cells and endothelial progenitor cells (EPCs) [41]. As the wound is virtually ischemia from the disruption of its vascular supply, CXCL12 aids in the response to ischemia by enhancing angiogenesis and vasculogenesis [42]. Additionally, CXCL12 may aid in the recruitment of mesenchymal stem cells from bone marrow reservoirs [43] and also enhance stem cell proliferation in bone marrow [41]. As such, it may be a promising target for engineered scaffolds, specifically those targeted for wound-healing applications [44]. CXCL12 also represents a fundamental signaling cue to bone marrow hematopoietic and circulating blood cells to move to the site of injury, thus prompting monocytes to enter injuries and begin their critical governance [44]. Macrophages may also influence levels of CXCL12 by secreting proteases which inhibit CXCL12, therefore reducing one of the wound’s most potent chemokines. As a result, macrophages are able to directly influence the transition from recruitment and inflammation to proliferation and remodeling through the CXCL12 signaling cascade [42]. 

In the second, proliferative phase of wound healing, macrophages, adhering to the ECM through integrin receptors, release colony-stimulating factor 1, platelet-derived growth factor (PDGF), IL-1, TGF-β, and TNF-α. These cytokines increase local inflammation and encourage differentiation from inflammatory to regenerative cell subtypes. Macrophages coordinate the transition to proliferative healing through regulation of fibroblast and keratinocyte responses [2,19]. The importance of macrophages was initially documented in 1975 through a series of anti-serum and steroid suppression experiments where, unlike neutrophils, impaired or depleted macrophages had a negative impact on the time to wound healing. After these initial experiments, subsequent data confirmed these findings and it is now widely recognized that macrophages function as a critical regulatory cell in wound healing [37,45,46].

The proliferative phase of wound healing is characterized not only by the differentiation of macrophages, but also by the formation of granulation tissue. Plasmin, plasminogen, and collagenase degrade ECM proteins and disrupt intercellular gap junctions and desmosomes to promote migration of keratinocytes from the wound edge into the wounded space. Behind the advancing front of migrating cells, additional keratinocytes expand and coalesce to create a new stratified squamous epithelium. Deeper in the dermis, which is now amalgamated with the epidermis after the dissolution of the ECM, fibroblasts proliferate and begin laying down temporary and permanent ECM structures. Vimentin and fibronectin are gradually replaced with type I collagen, which will serve as the ultimate scar substrate [2,28,47]. Additionally, the versican v3 isoform proteoglycan promotes the transition of fibroblasts into myofibroblasts, the cells responsible for collagen contraction [20]. Of note, there is significant heterogeneity between fibroblasts that has only recently been described [48]. While the presence of multiple distinct fibroblast populations was suggested in several studies, this was not definitively reported until 2015. Lineage tracing allowed the discovery of a solitary set of dermal fibroblasts that were responsible for the entirety of scar collagen deposition in murine dorsal skin [49,50,51]. Through this discovery, it is now understood that distinct fibroblast populations are responsible for scar formation and collagen deposition. Because of their functional differences, specific fibroblast populations may serve as a therapeutic target in the future, though this has not yet been reported.

Fairly noticeable in the newly formed granulation tissue are the many new blood vessels that assemble. As macrophages and fibroblasts secrete a number of growth factors, angiogenesis ensues. Eventually, the newly formed tissue has a much greater density of new vessels than uninjured skin. Hence, granulation tissue is given its characteristic reddish hue from budding capillaries. To clarify, angiogenesis is distinct from vasculogenesis. Vasculogenesis is the de novo formation of new vessels. By contrast, angiogenesis is characterized the creation of thin-walled endothelial tubes from preexisting blood vessels in the periphery and base of wounded tissue [12,18]. These new vessels supply another access point for recruited inflammatory cells, as well as improve oxygenation for collagen crosslinking and scar maturation [18].

In the third phase of wound healing, remodeling, the once cell-dense scar begins a process of apoptosis that removes all but a few fibroblasts and blood vessels. Additionally, the rapidly deposited collagen, formed in a parallel pattern compared to the basket-weave fibers seen in uninjured tissue, begins to contract. Myofibroblasts, signaled by deep dermal proteoglycans, aid in the establishment of collagen crosslinks. Additionally, mixed metalloproteases are simultaneously secreted by macrophages, keratinocytes, as well as fibroblasts to degrade collagen fibers that interfere with wound contraction. At the conclusion of the remodeling phase, scar tissue will have 70%–80% of its original tensile strength. The scar will not develop new melanocytes, pigment, sweat glands, or hair follicles [2,28,47]. 

Considering the inherent deficiencies of normally formed scar tissue compared with uninjured skin, it is not surprising that wounds with abnormal healing are a major burden to patients. Most dysfunctional wound healing is seen in chronic wounds or ulcerations, where, for a variety of reasons, a soft tissue injury will fail to close. Other disorders of the wound healing mechanism exist in hypertrophic scars. Hypertrophic scars have a dysfunctional apoptosis mechanism in the proliferative phase, and over-deposition of type I collagen. This eventually leads to a raised scar. Keloids, another dysfunctional scar type, are essentially a benign scar tissue tumor that continues to grow over time, often extending beyond the wound border. These examples of exaggerated wound healing are not only painful, but can cause physical limitations for patients through the development of contractures. This is in sharp contrast to an under-healing process, such as diabetic foot ulcerations, which can lead to major amputations, usually secondary to pain or infection [1,28]. Whether under- or over-healing, however, wound-healing disorders are costly and morbid.

Based on the cells, cell signaling cascades, proteins, and structures involved in wound healing, a number of potential targets to influence healing exist. Cell-based targets, such as corticosteroids, can reduce inflammation and potentially reduce resulting scars, albeit with a reduction in scar tensile strength [52,53,54]. In general, cell-based targets tend to have global wound healing influences or, due to redundancies, fail to achieve desired results. Targets of cell signaling cascades, such as TGF-β, IL-10, and PDGF, too, are redundant and involve multiple cell types, inevitably failing to achieve significant improvements in wound healing in humans [7,21,23,24,55,56]. As mentioned previously, it may be the extracellular matrix that ultimately regulates each of the recruited and proliferating cells in the wound bed [3,13,57,58,59]. Additionally, differences in the extracellular matrix between adults and fetuses may be the key between adult fibrosis and fetal scarless healing [60,61,62,63].

## 3. Biomaterials in Clinical Use

Most of the biomaterials currently in clinical use can be divided into a few categories: ECM substrates, decellularized matrices, and engineered stem cells. The clear majority of products coming into the market are ECM substitutes, highlighting the importance of the ECM in providing important cues for regenerative healing (Table 2). Biomaterials for wound healing are used in both acute and chronic scenarios, though with mixed results and unique benefits and drawbacks [47,64,65].

### 3.1. Scaffolds and ECM Substrates

#### 3.1.1. Collagen

Collagen is one of the most abundant structural proteins in vertebrate organisms. The majority of tissues in the human body are comprised of a combination of collagen subtypes, from types I to XX in humans, with types I–IV being the most prevalent [28]. Interestingly, the predominant collagen subtype in tissue may change with gestational age, as fetal studies have shown an abundance of type III collagen in fetal skin. Type III collagen then gradually shifts to type I collagen as the skin transitions from a regenerative to scarring phenotype. This phenomenon might be explained by the fibroblasts responsible for scar-collagen synthesis—otherwise known as *engrailed*-positive fibroblasts (EPFs) [28,51]. *Engrailed-1* is a homeodomain transcription factor expressed in early embryonic development in the dorsal and cranial mesenchyme. Through lineage tracing, a fibroblast population derived from *engrailed*-positive cells was found to be the sole fibroblast responsible for the synthesis of scar collagen in murine dorsal skin [51]. 

There is also a considerable amount of research in the area of fibril organization. While type I and III collagen fibers in unwounded skin are arranged in a basket-weave pattern, the same collagen in wounds is synthesized in parallel arrays [28,67]. Because of the characteristics of scar collagen deposition, the majority of bioengineered collagen scaffolds attempt to recreate the normal dermal or epidermal (or both) three-dimensional architecture. Their general concept is to serve as a scaffold to allow neovascularization and eventual ingrowth of keratinocytes in a more organized way, while also allowing early wound coverage [67,68,69]. The most significant clinical application of collagen scaffolds pertains to burn patients with large areas of dermal loss. Here, a skin substrate is beneficial in acting as a temporary closure, moisture and microbial barrier, and medium on which cultured epidermal cells may grow [63,67,70,71]. Unfortunately, clinical experience has shown poor engraftment results using collagen alone as a transplant substrate in in vivo studies, which may explain the transition to using collagen in cultured grafting techniques instead [72]. More research into the role of collagen in wound healing is needed before its translational effects are entirely realized.

#### 3.1.2. Fibrin

A component of the platelet plug often utilized as an adjunct for wound healing is fibrin. It is most commonly used as a surgical sealant and hemostatic agent, where it is quite effective. Fibrin, a product of the action of thrombin on fibrinogen, is the most abundant component of a platelet blood clot [28,73]. Like collagen, it is thought to enhance angiogenesis, granulation tissue formation, and fibroblast proliferation to promote wound healing [14,74]. Moreover, like hyaluronic acid, fibrin also serves as a substrate that allows inflammatory cells, keratinocytes, and fibroblasts to migrate through a wound bed. In the treatment of chronic and acute wounds, fibrin is often used as gel or sealant that can be combined with platelet-rich plasma to enhance the pro-regenerative qualities of a product [75].

One of the potential therapeutic benefits of fibrin is its ability to be enriched from blood products, making it an autologous source of ECM scaffold [76,77]. As a scaffold, fibrin could be used in patients to facilitate the ingrowth of regenerative cells. So far, the most promising clinical results have been in using fibrin as an autologous scaffold for the transplantation of cultured dermal fibroblasts in burn patients [14].

#### 3.1.3. Hyaluronic Acid

As one of the body’s major lubricating compounds, hyaluronic acid has emerged as a potential therapy to enhance wound healing [28]. This large glycosaminoglycan is most prevalent in synovial fluid and articular cartilage where it serves to absorb shock. It is also prevalent in the basement membrane and intercellular spaces between basal keratinocytes, where it augments diffusion of water and nutrients to supply epidermal stem cells. In addition, it is one of the major components of the extracellular matrix and platelet plug, promoting migration of cells through the wound bed via CD44 [78]. The role of hyaluronic acid (HA) in cell migration may explain some of its purported benefits in tissue regeneration and wound healing. However, it is also able to retain large volumes of water and scavenge free radicals, making it a popular choice for cosmetic procedures [9] where desiccation results in a poor outcome.

For promoting wound healing, hyaluronic acid is used as a topical pharmacologic agent [79]. It is readily absorbed into soft tissue, though its influence on the in vivo cellular environment is less certain. In recent articles, hyaluronic acid was used to enhance diabetic chronic wound healing through promotion of cell proliferation and angiogenesis. It was also used to aid in the closure of complicated Achilles tendon reconstruction with major soft tissue defects [80,81]. However, there appear to be important distinctions between HA compounds of different molecular weights. While high molecular weight HA has anti-inflammatory effects, low molecular weight HA is a potent proinflammatory compound [78]. This could be the reason for mixed clinical results, as earlier studies may not have taken these facts into account. Ultimately, promising in vitro studies on the proliferative, angiogenic, and anti-inflammatory properties of HA have failed to provide a significant impact on chronic or acute wound healing in vivo [82].

#### 3.1.4. Chitosan

Vertebrate ECM substrates are not the only scaffolds currently in use. Chitosan, a linear polysaccharide derived from cleavage of chitin, comes from the exoskeleton of insects, shellfish, and the cell wall of some fungi. Usually this compound is isolated from shrimp shells and is then chemically treated to produce a more malleable material. The resulting product can be used for 3-D printing or creating porous scaffolds that are both biocompatible and biodegradable [28,83]. For patients, the product has potential as a vulnerary agent due to its rapid incorporation and eventual dissolution in human tissues. Additionally, chitosan has promise as a drug delivery agent, because the scaffold can be impregnated to deliver steady concentrations of pharmaceuticals [84,85]. However, chitosan has some concerning toxic effects, namely dermal inflammation seen in certain doses of consumed chitosan. This could limit the eventual potential of this product [85].

### 3.2. Acellular Dermal Matrices

An alternative to single protein/glycoprotein ECM substitutes is to use a decellularized dermal matrix. These products—such as Surgimend, FlexHD, and Alloderm—are well known to general and reconstructive surgeons for their ability to serve as meshes and closure materials in areas where significant tissue was lost [86]. Originally introduced to the surgical community in 1994 in the context of burn reconstruction, the market for decellularized dermal matrices has grown substantially, along with their applications in surgery [87]. These porcine- and human-derived ECM substrates are unfortunately expensive and carry a risk of rejection, infection, and allergy for their recipient (usually donated from either a cadaveric human or pig source). However, like other ECM substitutes, acellular dermal matrices (ADMs) can offer a protective barrier, a scaffold through which cells may migrate, and cell signaling matrix to promote angiogenesis and granulation tissue ingrowth [87,88]. The most promising substrates, in the process of decellularizing, retain most of the ECM superstructure, like hyaluronic acids, proteoglycans, and glycosaminoglycans.

The biggest market for ADMs is in abdominal wall and breast reconstruction (Table 2), where the dermal tissue is used to provide structural support [89,90]. In wound healing, however, ADMs fail to provide enough clinical impact to warrant their frequent use. In large part, this is due to their cost and the need for native cells to infiltrate the wound bed. Some products, namely xenograft materials, also require eventual excision after granulation tissue develops. This subjects patients to more surgery and potentially longer hospital stays. Their use, too, has not reduced the need for autologous engraftment, and the purported ability of ADMs to enhance split-thickness skin graft survival is dubious [91].

Unfortunately, ADMS are not often compared in randomized clinical trials head-to-head. As such, there are a variety of different products that are United States Food and Drug Administration (FDA) approved and have proven efficacy, but their comparative differences are unknown. Boyce et al. have compared several products; highlighting their differences and the importance of selecting ADMs specifically for individual patient needs [92]. For instance, full thickness burn injuries covering large surface areas may benefit from an ADM that contains dermal and epidermal components, as well as cultured cells [92]. In full thickness dermal injuries that cover less extensive areas, cografting may be a method of regenerating dermis and epidermis. One study achieved excellent results using an ADM to generate dermal architecture, and then a split thickness skin graft some days after the ADM incorporated into the wound [93]. In breast and abdominal wall reconstruction, ADMs from human skin are preferred due to their strength [87,91]. However in wound healing, products such as the amniotic membrane-derived BIOVANCE may have an more dramatic impact, since it contains both fetal architecture and important ratios of hyaluronic acid, TGB-βs, and collagens [94].

Lastly, several products have recently fallen under scrutiny due to their procurement methods [95]. As a clinician, the use of ADMs should be limited to specific patients—namely, those with particularly difficult-to-treat wounds who have exhausted cost-effective methods. Partly, this is due to the cost of ADMs, but also because of their mixed clinical results. On the whole, clinical trials in wound healing with ADMs have low power, small sample sizes, and fail to compare products head-to-head with the exception of a few examples [88,90,96,97].

## 4. Experimental Biomaterials and Future Directions

Emerging products to treat wound healing often incorporate a combination of ECM compounds, three-dimensional architecture, and cell therapy. Products like Hyalograft combine cultured ECM matrix with autologous cartilage cells to regenerate lost tissue [28]. Other products aim to recapitulate the fetal wound-healing environment, such as BIOVANCE and EpiFix. Both are human amniotic membrane allografts that claim to be capable of improving wound healing, time to closure, and also confer some immune protection for grafted cells [94,98]. Synthetic biodegradable polymers are also an emerging topical wound therapy. These are usually bimembranes, with a hydrogel on one side that can be applied directly to the wound. On the other membrane is a water-impermeable product such as silicon that can provide a protective barrier to the wound environment to facilitate healing [99]. Most of these gradually degrade via hydrolysis into nontoxic substances that are easily metabolized [100]. One of the advantages of polymer-based therapies is that they offer no infection or allergy risk to the patient. Also, their cost can be markedly reduced through more efficient synthesis techniques and economies of scale [101,102].

Therapy with adult stem cells has also gained popularity since the discovery of niche-specific stem cells. Adipose-derived and mesenchymal stem cells have promising in vitro and in vivo studies showing their ability to form new blood vessels in transplanted tissue and accelerate time to wound healing. Mesenchymal stem cells (MSCs) are theoretically available in a number of human tissue reservoirs, but are most commonly isolated from bone marrow (BM) [103,104]. Due to the cellularity of extracted marrow, BM-MSCs require expansion in culture [105,106]. FDA approval is more challenging when using cultured cells, particularly if not autologous, because of the risk of contamination and infection. This aspect of MSC derivation may limit their clinical potential. Adipose-derived stromal cells (ASCs) have, perhaps, greater clinical promise. They are abundant in adipose tissue, can be easily harvested with little donor morbidity, and have the potential to differentiate into a variety of tissue types [107]. Because of the abundance of ASCs, donation could be autologous and would not require ex vivo expansion. However, both ASCs and MSCs require additional materials to facilitate engraftment [108]. Delivery with a scaffold or hydrogel improves cell delivery and time to wound closure. However, the number of cells necessary to achieve improved wound healing per patient is not yet well established. Therefore, while precise tools and delivery methods may be available, their clinical use is still largely theoretical. Additionally, the ASC’s purported ability to differentiate into a number of mesenchymal and ectoderm-based cells needs further study [109]. Ultimately, the age and comorbidities of the donor may have a significant impact on the ASC’s effectiveness as a cell-based therapy.

Lastly, two other sources of adult autologous stem cells are currently under development. Hair follicle bulge cells, along with several other hair follicle-derived stem cells, are known to differentiate into keratinocytes and participate in early re-epithelialization of skin wounds [47,110]. In a similar pattern to keratinocytes, they migrate in a radial pattern into the center of a wound. There, they proliferate to create a stratified squamous epithelium [28]. However, more recent evidence suggests their contribution to wound healing is temporary, and inevitably the cells disappear from the scar architecture once healed [110]. Because of their role in early wound healing, it is therefore not surprising that hair follicle stem cells may represent a reasonable method of autologous cell engraftment [111], but, like ASCs and BM-MSCs, require a surgery for procurement. Skin-derived precursor cells (SKPs) can also differentiate into glial, epidermal, and dermal cell types [112]. SKPs then, too, could be used as a type of cell-based wound therapy [113,114,115]. In fact, there may be multiple sources of precursor cells within the hair follicle that contribute to wound healing [116]. Portions of the hair follicle, from its local blood supply, interfollicular epidermal cells, and associated mast cells and macrophages, appear to be actively recruited into the wound bed to directly influence wound healing [116]. Recently, Surrao et al. developed a method of cell culture that rapidly expands SKP cells in a controlled environment [112]. While this represents an exciting step toward scale-up of stem cell therapies, transplantation of cultured SKPs in this scenario would be allogenic and therefore subject to eventual graft rejection.

Engraftment rejection is an important barrier to the use of stem cells in adults. Whereas a fetus is capable of chimerism, adults are not. MSCs and ASCs could avoid the issue of immune rejection, as they can be harvested locally in vast quantities. While their procurement is morbid, autologous stem cell transplantation is clearly preferred if utilized in human patients. Partly, this is because autologous transplantation would allow long-term engraftment and influence in the wound environment [108,117,118]. However, in those with chronic medical conditions, surgical procurement may not be possible if the patient’s health does not permit surgical intervention. In those cases, allogenic stem cell transplantation is possible, but may require manipulation in culture to reduce rejection. Alternatively, rejection may not be an issue if allogenic stem cells provide cues to influence wound healing in their initial engraftment period. For instance, applying MSCs or ASCs in the first four days of acute wound healing may be adequate to improve the time to wound closure and reduce scar formation. After that time, their destruction by the host immune system may be acceptable. In the case of keratinocytes, or hair follicle bulge cells, procurement of enough cell numbers to influence chronic or acute wound healing may be difficult. As such, even with autologous transplantation, it may be impossible to obtain adequate cell numbers to influence wound healing [66,103,111]. With rapid cultured expansion, other dermal stem cell precursors could be available as allogenic stem cell therapies [108,111,119].

## 5. Discussion

Through this review, the major biomaterials currently in clinical and experimental use were investigated and discussed. As it appears now, the role of biomaterial fabrication tends to focus on creating extracellular matrix-like scaffolds to facilitate skin healing through provision of normal three-dimensional tissue architecture. While these products may offer a clinical benefit to patients, they do not offer dramatic results and require the ingrowth of a variety of cell types that may be either defective, such as in patients with chronic nonhealing wounds, or lost, as is the case in burns and major soft tissue injury. Additionally, these products depend on the adult wound-healing mechanism, which is arguably imperfect. To obtain truly regenerated dermal tissue, focus needs to be turned to the inherent differences between adult and fetal wound healing. Attempts should be made to recapitulate the fetal environment for cell recruitment, differentiation, and tertiary structure formation. For instance, major developmental signaling molecules such as Wnt (wingless protein) reveal a role for using embryonic signals in adult wound healing. Wnt3a and fibroblast growth factor 9 (FGF9), both present in some quantities in adult tissue, are being developed as vulnerary agents capable of regenerating dermal appendages in adult scars [50,120,121].

Again, the main goal of wound-healing study and product development is to speed the time to wound healing in chronic wounds and acute wounds, and to reduce scar formation. The only example of regenerative healing exists in fetal wounds, which, prior to a certain gestational age, will regenerate completely and leave no evidence of scar. With the exception of a few examples, most wound-healing products mimic adult differentiated tissue rather than the fetal environment. While this approach can produce an improved cosmetic and functional outcome through recruitment of skin progenitor cells and dampening of the immune system, dermal appendages will not regenerate. Additionally, to recreate the fetal wound-healing environment, three-dimensional scaffolds containing both dermal and epidermal structures, as well as dermal appendages, are likely necessary for skin to regenerate nerves, hair follicles, sebaceous glands, and other important skin structures. Therefore, products that contain single ECM proteins may eventually be limited to roles in pharmaceutical delivery, knowing they are not otherwise capable of regenerating skin.

Hopefully, through the investigation of fetal wound healing, new products can achieve tissue regeneration without sacrificing the speed of adult wound healing. Healed tissue could then be functionally similar to normal skin, and therefore less likely to break down, recur, or become hyperplastic. Ultimately, a more elastic and less fibrotic outcome will lead to better physical and psychological outcomes for our patients.

## 6. Materials, Methods and Supplemental Information

Articles were reviewed independently by the authors. The following databases were utilized: Medline, JSTOR, Google Scholar, PubMed, and SCOPUS. Articles were excluded if they did not involve skin, such as adipose tissue, bone, tendon, or nerves. Articles were excluded if they did not utilize cellular or engineered materials for the purposes of wound healing. External devices were not evaluated.

## Figures and Tables

**Table 1:** Inflammatory cytokines and their impact on wound healing *.

Inflammatory Cytokine	Role in Inflammation	Clinical Considerations
CXCL12	Increase inflammation, EPC recruitment, leukocyte recruitment	May improve time to wound healing in CXCL12 impregnated scaffolds
EGF	Keratinocyte proliferation and mobilization	Can be impregnated into synthetic matrices
FGF	Fibroblast and keratinocyte proliferation, migration	ASCs with high expression of FGF may enhance endothelial differentiation in wounds
IL-10	Reduces inflammation, transition to regenerative healing	May be important for scarless fetal healing
M-CSF	May stimulate local stem cell proliferation	Accelerates time to wound healing if applied topically in sickle cell patients
Monocyte chemoattractant-1	Recruitment of circulating monocytes, monocyte differentiation	Can be downregulated through TNF-α inhibition
NO	Regulates collagen deposition, wound contraction. Vasodilatory	Impairs DNA synthesis, therefore may lead to impaired healing if used as a vulnerary agent
PDGF	Macrophage recruitment, fibroblast proliferation	Accelerates wound healing in diabetics, chronic pressure ulcers, and venous stasis ulcers
TNF-α	Inflammatory cell recruitment	May impair fibroblast proliferation and collagen deposition
VEGF	Promotes angiogenesis and granulation tissue formation	Expression is controlled by cell surface marker CD44

* Table includes data from the following references [2,18,26,27,28,29,30,31,32,33,34,35,36]. Abbreviations: CXCL12, C-X-C motif chemokine ligand 12; EGF, endothelial growth factor; FGF, fibroblast growth factor; IL-10, interleukin-10; M-CSF, monocyte colony-stimulating factor; NO, nitric oxide; PDGF, platelet derived growth factor; TNF-α, tumor necrosis factor-α; VEGF, vascular endothelial growth factor.

**Table 2:** Clinically relevant biomaterials for wound healing *.

Substrate	Origin	Composition	Clinical Examples
*ECM Substitutes*	Animal or plant tissue	Single to multilayer films with variable 3-D architecture	Beriplast, Biocol, Hyaff, Hycoat, Hyalomatrix, Promogran, Puraply
*Acellular Dermal Matrices*	Porcine or Human	Decellularized skin, chemically treated to achieve sterility and improve tensile strength	AlloDerm, DermaSpan, FlexHD, Surgimend
*Synthetic Compounds*	Electrospinning, salt lithography, soft lithography stamping, solid free-form fabrication	Single and multi-compound materials, can be impregnated with pharmaceuticals	Biobrane, Dermagraft, Integra
*Cultured Stem Cells*	Human amniotic membrane, dermal fibroblasts, BM-MSCs, ASCs, neonatal prepuce	Most often cells are embedded or seeded onto cultured scaffolds	Apligraf, denovoSkin, denovoDerm, Epicel, EpiFix, OrCel, StrataGraft, Transcyte, Triscover

* Table includes data from the following resources: [60,66]. Abbreviations: MSC, mesenchymal stem cell; ASC, adipose-derived stromal cell

## Abbreviations

The following abbreviations are used in this manuscript:

ACM	acellular dermal matrix
ASC	Adipose-derived stromal cell
BM-MSC	Bone marrow mesenchymal stem cell
CXCL12	C-X-C motif chemokine ligand 12
ECM	Extracellular Matrix
EGF	Endothelial growth factor
EPF	Engrailed-positive fibroblast
FDA	United States Food and Drug Administration
FGF	Fibroblast growth factor
HA	Hyaluronic acid
IL-#	Interleukin-#
M-CSF	Monocyte colony-stimulating factor
MSC	Mesenchymal stem cell
NO	Nitric oxide
PDGF	Platelet derived growth factor
SKP	Skin-derived precursor cell
TGF-β	Transforming growth factor-β
TNF-α	Tumor necrosis factor-α
VEGF	Vascular endothelial growth factor
Wnt	Wingless protein
